# Clinical features and outcomes of horses presenting with presumed equine immune mediated keratitis to two veterinary hospitals in the United Kingdom and Finland: 94 cases (2009–2021)

**DOI:** 10.1111/evj.14213

**Published:** 2024-08-26

**Authors:** Juliette F. Preston, Minna P. Mustikka, Simon L. Priestnall, Bettina Dunkel, Maria‐Christine Fischer

**Affiliations:** ^1^ Department of Clinical Science and Services Royal Veterinary College Hatfield Hertfordshire UK; ^2^ Department of Equine and Small Animal Medicine University of Helsinki Helsinki Finland

**Keywords:** cornea, corneal ulcer, eye, horse, IMMK, inflammatory, ocular, ophthalmic, pain

## Abstract

**Background:**

There is limited literature regarding equine immune mediated keratitis (IMMK) in Europe. North America‐based publications describe minimal blepharospasm, rare corneal ulceration and no uveitis; clinical impression suggests these are seen in Europe.

**Objectives:**

Assess the prevalence of blepharospasm, corneal ulceration and uveitis and their impact on outcome in horses diagnosed with IMMK in Europe (UK and Finland).

**Study design:**

Retrospective case series.

**Methods:**

Clinical records of 94 horses with IMMK were evaluated. The UK and Finland populations were comparable; therefore, descriptive statistics were performed on combined data on subtypes of IMMK and clinical features. Odds ratios (OR) and confidence intervals (CI) were calculated for impact of blepharospasm, ulceration or presence of uveitis on the outcome of enucleation and treatment duration.

**Results:**

IMMK subtype was classified as 10/94 (10.6%) epithelial, 50/94 (53.2%) anterior stromal, 14/94 (14.9%) mid‐stromal, 4/94 (4.25%) endothelial and 16/94 (17.0%) unrecorded. After excluding three horses with incidental corneal ulceration, blepharospasm was documented in 34/91 (37.4%), corneal ulceration in 26/91 (28.6%), and signs of uveitis in 23/91 (25.3%) horses. Increased odds of enucleation were significantly associated with the presence of blepharospasm (OR 5.5, 95% CI 1.6–19.4, *p* = 0.008) and signs of uveitis (OR 8.9, 95% CI 2.6–30.8, *p* < 0.001), but not corneal ulceration. The presence of blepharospasm, corneal ulceration or uveitis did not significantly alter the odds of ongoing medication.

**Main limitations:**

Data were collected over a wide timeframe; the diagnosis was mainly made without histopathology; a broad definition of uveitis was used and there was a bias towards complicated cases being retained for follow‐up.

**Conclusions:**

The clinical features of IMMK were similar between two European countries but differed to USA descriptions. Blepharospasm, corneal ulceration and signs of uveitis can occur with IMMK; presence of blepharospasm and uveitis increase the odds of enucleation.

## 1 INTRODUCTION

**FIGURE 1:**
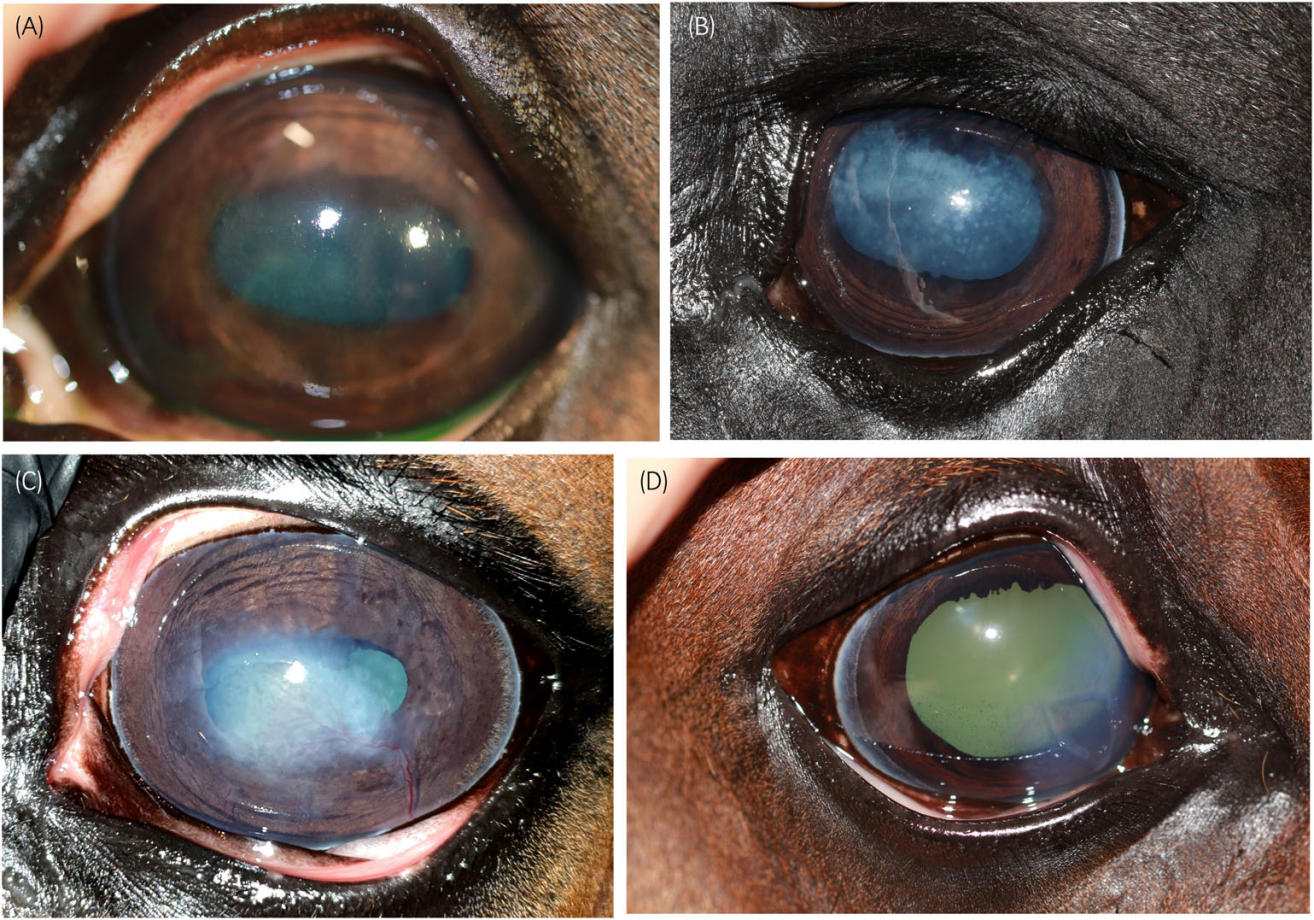
(A) Epithelial immune mediated keratitis (IMMK) with erosive fluorescein uptake, (B) anterior stromal IMMK, (C) midstromal IMMK and (D) endothelial IMMK with superficial corneal ulceration evident. Image D displays an indolent type of corneal ulcer, as can be found secondary to marked corneal oedema. With thanks to Roser Tetas for images A, B and D, and Minna Mustikka for image C.

Immune mediated keratitis (IMMK) is typically described as chronic, non‐ulcerative corneal opacity with minimal discomfort in the absence of intraocular inflammation.^1^, ^2^, ^3^ Subtle differences have been acknowledged between cases from the United Kingdom (UK) and the United States of America (USA).^3^ IMMK has been divided into four subtypes: epithelial, anterior stromal (chronic superficial keratitis in some veterinary literature^1^), mid‐ to deep‐stromal (chronic recurrent keratitis in some veterinary literature^1^) and endothelial (Figure 1).^1^, ^2^, ^3^ Some literature merges the epithelial and anterior stromal form into one ‘superficial’ form.^4^, ^5^ The description of discomfort varies between the corneal depth classifications and the geographical location. In the USA, all forms of IMMK are generally considered nonpainful apart from mild discomfort occasionally seen in the epithelial subtype. In the UK, the epithelial and anterior stromal forms have been inconsistently associated with slight blepharospasm.^3^ Corneal ulceration is rarely described in either geographic location, but is reported occasionally in the mid‐stromal and endothelial forms in both countries associated with ruptured corneal bullae, which in turn can cause discomfort.^3^ It is challenging to determine if the presence of corneal ulceration is attributed to the initial disease process, or the effect of secondary degenerative changes such as corneal mineralisation which could be due to chronicity of IMMK or to the use of long‐term topical corticosteroid preparations.^4^, ^6^ To the authors' knowledge, uveitis has not been described as a clinical feature of IMMK.

IMMK is considered an inflammatory condition given its histological picture and response to topical corticosteroids and calcineurin inhibitors.^3^, ^7^, ^8^, ^9^, ^10^, ^11^ Reported response to subconjunctival mesenchymal stem cell therapy further supports an inflammatory cause.^12^ A previous report investigating the histopathological features of the disease identified lymphoplasmacytic cellular infiltration, and immunohistochemical investigations support a CD3+ T‐cell mediated response.^11^ In vivo confocal microscopy has demonstrated that the anatomic location of hyperreflective pathology correlates well with the clinical classification. Additionally, numerous antigen‐presenting cells were identified in the epithelial basement membrane region and immediate subepithelial corneal stroma in all clinical classifications of IMMK.^13^ Attempts to identify a corneal autoantibody causing chronic antigenic stimulation have had limited success.^14^ It has been speculated that the difference between UK and USA cases might be attributed to a variation in causative agents of infectious keratitis resulting in secondary chronic antigenic stimulation, but supporting literature is lacking.^3^

IMMK commonly requires long‐term medication, typically with topical immune‐modulatory drugs.^3^, ^8^, ^9^ Challenges associated with this treatment are (1) patient compliance for administration, (2) costs of long‐term treatment, (3) compliance with rules regarding performance enhancing drugs for use in competitions, and (4) variable treatment response of the different subtypes of IMMK.^4^, ^5^, ^15^ All of these factors might impact decision making when considering different treatment options. In addition, use of long‐term topical immunomodulatory medication has been associated with corneal degeneration, corneal stromal abscesses, protozoal infections and fungal keratitis.^6^, ^16^, ^17^, ^18^, ^19^

In the authors' experience, blepharospasm, corneal ulceration and uveitis can be present as part of the clinical picture of IMMK, which is inconsistent with some of the previous literature. The aims of this project were first to identify if the disease had similar clinical features in the UK and Finland, two geographically distinct Northern European countries, and second, to combine the datasets to determine the frequency of ocular discomfort, corneal ulceration and uveitis and the impact of those on the outcome.

## 2 MATERIALS AND METHODS

The clinical database for both the Royal Veterinary College, University of London and the Faculty of Veterinary Medicine, University of Helsinki were searched for cases with a diagnosis of IMMK. All patients included had a full ophthalmic examination performed by either a Diplomate or a resident‐in‐training of the European College of Veterinary Ophthalmologists, or a Diplomate of the European College of Equine Internal Medicine. A diagnosis of IMMK was made based on some or all of the following: patient history, clinical findings on ophthalmic examination (including tonometry, slit lamp biomicroscopy, direct and/or indirect ophthalmoscopy, fluorescein stain uptake), corneal cytology and favourable response to treatment with immunomodulatory agents. To remove intrinsic bias within the dataset only one eye of each horse that presented with bilateral disease was included. If both eyes had chronologically asymmetric disease, the eye with the longest duration of clinical signs was included. Those that had equal duration of signs reported had the eye to be included chosen using a randomiser website (GraphPad QuickCalcs©). Horses were excluded if incomplete ophthalmic examination findings were recorded, if there was intraocular disease suggestive of equine recurrent uveitis or any other intraocular disease that could cause uveitis.

For each enrolled case signalment (age, breed, sex), duration of clinical signs and eye affected were noted. Recorded ophthalmic findings at the first consultation were blepharospasm, ocular discharge, conjunctival hyperaemia, corneal oedema, corneal infiltrates and corneal neovascularisation. Where the clinician for the case had indicated a specific depth of the focus of corneal pathology, this was used to classify the IMMK subtype as epithelial, anterior stromal, mid‐stromal or endothelial. Additionally, presence of corneal ulceration was documented and if the ulceration appeared consistent with the IMMK pathology or was considered unrelated (e.g., trauma in transit). If corneal ulceration was identified and not considered to be a part of the pathology of IMMK, then these cases were not included in further statistical analyses regarding blepharospasm, corneal ulceration or presence of uveitis signs. Features of possible uveitis were recorded, including presence of miosis (based on clinical examination without pupillometry), aqueous humour flare and low intraocular pressure (IOP). If atropine had been applied prior to assessment resulting in a dilated pupil this was noted. Intraocular pressure was measured using the Tonovet® (iCare, Finland) in both locations and low IOP was defined as an IOP ≥6 mmHg lower than in the healthy fellow eye, this was not determined for eyes without a healthy fellow eye for comparison.^20^ A ‘normal’ reference range for intraocular pressure was not used, as the intraocular pressure is affected by time of day, type of tonometer, use of topical anaesthesia, use of atropine, administration of an auriculopalpebral nerve block, sedation, type of drugs used for sedation as well as head position^21^, ^22^, ^23^, ^24^, ^25^, ^26^, ^27^, ^28^, ^29^, ^30^, ^31^; these details were not consistently available in the clinical records and therefore could not be adjusted for. Horses were classified as having signs of uveitis if at least one of the following findings was present: low IOP, aqueous flare or miosis. The medication that the patient was receiving at the time of examination and medication prescribed after examination was documented including episcleral ciclosporin A implants. Patients that received episcleral ciclosporin A implants while under the care of the RVC or Helsinki hospitals had them placed according to previously described techniques.^9^

Any ocular surgery or euthanasia and reason for this performed at the time of examination or at any time during follow‐up was noted. Follow‐up information included the duration of follow‐up, ocular comfort and vision, presence of corneal scarring and whether medication was ongoing. The degree of corneal scarring was classified as ‘mild’ or ‘moderate’ based on the investigators' interpretation of the clinical records; at the outset a ‘severe’ category was planned but no cases were identified.

Data analysis was performed using SPSS 28.0. The normality of distribution of all continuous data was assessed with a Shapiro–Wilk normality test supported by histogram interpretation. Data were expressed as mean ± standard deviation for normally distributed, as median and range (minimum to maximum) for not normally distributed data and as numbers and percentages for categorical data.

A comparison between populations was performed for the following data; breed, sex, age at first presentation, duration of clinical signs, eye affected, presence of blepharospasm, discharge, hyperaemia, corneal oedema, corneal infiltrate and corneal neovascularisation, IMMK classification, presence of uveitis (low IOP, aqueous humour flare, miosis), presence of corneal ulceration, duration of follow‐up, the time to end‐point of the study (euthanasia, enucleation, discontinuation of treatment or lost to follow‐up), the presence of euthanasia or enucleation as the final outcome and the presence of vision, scarring and comfort at the final examination using a Pearson's Chi Squared test or Fischer's Exact test for categorical data, and a Mann Whitney *U* test for continuous data (Table 1). The degree of scarring at final examination was subjectively classified as mild, moderate or severe based on interpretation of the clinical report. As the two populations were found to be similar, the information was combined for further statistical analysis.

**TABLE 1:** This table outlines the differences in the signalment, presenting signs, clinical outcome and follow up between the RVC and Helsinki populations.

	RVC		Helsinki		Statistical test and test value	*p* Value
Breed classification						
Warmblood	21/43 (48.8%)	Irish Sport Horse (*n* = 8) Dutch Warmblood (*n* = 4) Hanovarian (*n* = 2) Andalusian (*n* = 1) Holstein (*n* = 1) Oldenburg (*n* = 1) Trakehner (*n* = 1) Unspecified Warmblood (*n* = 1) Warmblood Cross (*n* = 1) Westphalian (*n* = 1)	37/51 (72.5%)	Standardbred (*n* = 15) Warmblood (*n* = 11) Finnish Warmblood (*n* = 6) Trakehner (*n* = 3) Holstein (*n* = 1) Westphalian (*n* = 1)	Fisher‐Freeman‐Holton Exact	**<0.001**
Native small breeds	11/43 (25.6%)	Welsh Pony (*n* = 5) Dartmoor Pony (*n* = 1) Highland Pony (*n* = 1) Polo Pony (*n* = 1) Pony (*n* = 1)	5/51 (9.8%)	Pony (*n* = 4) Icelandic Horse (*n* = 1)		
Thoroughbred and Thoroughbred crosses	5/43 (11.6%)	Thoroughbred (*n* = 4) Thoroughbred Cross (*n* = 1)	0			
Draught horses and draught horse crosses	4/43 (9.3%)	Cob (*n* = 1) Friesian (*n* = 1) Irish Draught (*n* = 1) Irish Draught Cross (*n* = 1)	9/51 (17.6%)	Finnhorse (*n* = 8) Clydesdale (*n* = 1)		
Unrecorded	3/43 (7.0%)		0			
Sex						
Gelding	25/43 (58.1%)		24/51 (47.1%)		Fisher‐Freeman‐Holton Exact	0.3
Mare	15/43 (34.9%)		26/51 (51.0%)			
Stallion	2/43 (4.7%)		1/51 (2.0%)			
Unrecorded	1/43 (2.3%)					
Age						
Median (range) years	11.5 (4–31)		10 (1–28)		Mann Witney *U* *U* = 1220.5, *z* = 1.794	0.07
Duration of signs						
Median (range) weeks	7 (0.5–312)		6 (1–364)		Mann Witney *U* *U* = 844.5 *Z* = 1.012	0.3
Eye						
Right	20/43 (46.5%)		25/51 (49.0%)		Pearson's Chi Squared *χ* ^2^ = 0.059, df = 1	0.8
Left	23/43 (53.5%)		26/51 (51.0%)			
Bilateral	4/43 (9.3%)		7/51 (13.7%)			
IMMK classification						
Epithelial	4/43 (9.3%)		6/51 (11.8%)		Pearson's Chi Squared (excluding the cases that were unrecorded)	0.9
Anterior stromal	16/43 (37.2%)		34/51 (66.7%)			
Mid‐stromal	5/43 (11.6%)		9/51 (17.6%)			
Endothelial	2/43 (4.7%)		2/51 (3.9%)			
Unrecorded	16/43 (37.2%)					
Signs of uveitis (excluding three cases with non‐IMMK ulcers)						
Miosis	7/41 (17.1%) 7/38 (18.4%) excluding cases with atropine		7/50 (14.0%) 7/40 (17.5%) excluding cases with atropine		Fisher‐Freeman‐Holton Exact Pearson's Chi Squared *χ* ^2^ = 0.011, df = 1	0.3 0.9
Flare	4/41 (9.8%)		1/50 (2.0%)		Fisher's Exact Test	0.2
Low IOP (of cases where data available)	4/30 (13.3%)		8/43 (18.6%)		Pearson's Chi Squared *χ* ^2^ = 0.357, df = 1	0.8
Miosis, flare or low IOP	10/41 (24.4%)		12/50 (24.0%)		Pearson's Chi Squared *χ* ^2^ = 0.02, df = 1	>0.9
Follow up						
Median (range) days	19 (0–1061)		31 (0–1430)		Mann Witney *U* *U* = 1020.5 *Z* = 0.781	0.4
(*n*) horses with follow‐up available	32		34		‐	‐
(*n*) of horses with final ophthalmic examination	20		27		‐	‐
Enucleation						
(*n*) of horses with follow‐up	8/32 (25.0%)		6/34 (17.6%)		Pearson's Chi Squared *χ* ^2^ = 0.533, df = 1	0.6
Enucleation and euthanasia						
(*n*) of horses with follow‐up	12/32 (37.5%)		7/34 (20.6%)		Pearson's Chi Squared *χ* ^2^ = 2.30, df = 1	0.2
Final exam findings						
Visual	20/20 (100%)		27/27 (100%)		‐	‐
Comfortable	17/20 (85.0%)		26/27 (96.3%)		Fisher's Exact	0.3
Scarring	8/20 (40.0%)		21/27 (77.8%)		Pearson's Chi Squared *χ* ^2^ = 9.199, df = 1	**0.02**

*Note*: The bold font indicates the *p* value is considered significant and is below 0.05.

Pearson's Chi Squared test was used to determine if there was an association between each of; blepharospasm, corneal ulceration and signs of uveitis. In addition, Pearson's Chi Squared test was used to evaluate for an association between the outcome of enucleation and the presence of blepharospasm, corneal ulceration or signs of uveitis. Odds ratios were calculated for the impact of blepharospasm, corneal ulceration or presence of uveitis on the outcome of enucleation for all cases (excluding incidental ulcers) and the need for ongoing medication and presence of vision at last re‐examination for those with follow‐up available.

## 3 RESULTS

Information from 43 horses presenting to the RVC from January 2009 to November 2021, and 51 horses presenting to the University of Helsinki from February 2017 to March 2021 were included. Three of the RVC cases included had presented prior to formation of a specialised ophthalmology service and were diagnosed by Diplomates of the European College of Equine Internal Medicine.

The only significant differences identified between the signalment, presenting features and follow‐up of the two populations were breed distribution (*p* < 0.001) and the presence of scarring at the last re‐examination (*p* = 0.018; Table 1). The data were therefore combined for further statistical evaluation.

The presence of the following ophthalmic abnormalities was recorded using the combined data for all 94 horses; blepharospasm (35/94, 37.2%), ocular discharge (22/94, 23.4%), conjunctival hyperaemia (36/94, 38.3%), corneal oedema (63/94, 67.0%), corneal infiltrate (78/94, 83.0%) and corneal neovascularisation (65/94, 69.1%). A total of 16/94 (17.0%) horses had received atropine prior to referral, 14 of the remaining 78 horses (17.9%) displayed miosis. A qualitative description was given in 12/14 eyes with miosis; ‘mild’/‘minimal’/‘slightly’ (*n* = 10), ‘moderate’ (*n* = 1) and ‘marked’ (*n* = 1). All horses that were classified as miotic still retained a direct pupillary light reflex. Aqueous humour flare was present in 6/94 (6.4%) horses and a qualitative description was given in all eyes; ‘trace’/‘very mild’ (*n* = 3), ‘mild’/‘minimal’ (*n* = 2), ‘moderate’ (*n* = 1). Corneal ulceration was identified in 29/94 (30.9%) horses; in three cases, this was considered to be due to trauma in transit and not associated with the IMMK pathology. Of the three horses with corneal ulceration not associated with IMMK, one displayed blepharospasm, an atropinised pupil and trace aqueous humour flare; one displayed no blepharospasm, an atropinised pupil and no aqueous humour flare, and the third had no blepharospasm, a normal pupil and no aqueous humour flare. All three cases were subsequently excluded from further statistical analysis regarding blepharospasm, uveitis and corneal ulceration. A comparison of the IOP between the affected and fellow eye was available in 73 horses, with 12/73 (16.4%) classified as ‘low’. The median IOP of the fellow eye was 18.0 mmHg (range 9–41 mmHg), and that of the affected eye was 15.5 mmHg (range 8–35 mmHg), with a mean reduction in IOP of 1.9 mmHg (±4.8 mmHg), *p* = 0.001. A total of 22/91 (24.2%) of horses (excluding those with presumed transport associated corneal ulceration) showed signs of uveitis (miosis, aqueous humour flare or low IOP). Of those horses that displayed any signs of uveitis 11/22 (50.0%) did not have corneal ulceration documented. The signs of uveitis displayed by the 11 cases that did not have corneal ulceration included; low IOP (*n* = 6), unclassified miosis (*n* = 1), ‘mild’/‘slight’ miosis (*n* = 6) and ‘minimal’ flare (*n* = 1). Table 2 demonstrates the frequency of blepharospasm, corneal ulceration and signs of uveitis for each IMMK subtype.

**TABLE 2:** This table summarises the frequency of each subtype of IMMK and the frequency of blepharospasm, corneal ulceration and uveitis within each subtype.

IMMK subtype	Total number	Blepharospasm (excluding three cases with non‐IMMK ulcers)	Corneal ulceration (excluding three cases with non‐IMMK ulcers)	Uveitis (excluding three cases with non‐IMMK ulcers)
Epithelial	10/94 (10.6%)	3/10 (30.0%)	4/10 (40.0%)	2/10 (20.0%)
Anterior stromal	50/94 (53.2%)	18/50 (36.0%)	15/50 (30.0%)	11/50 (22.0%)
Mid‐stromal	14/94 (14.9%)	5/13 (38.5%)	2/13 (15.4%)	4/13 (30.8%)
Endothelial	4/94 (4.25%)	3/3 (100%)	1/3 (33.3%)	2/3 (66.7%)
Unclassified	16/94 (17.0%)	5/15 (33.3%)	4/15 (26.7%)	3/15 (20.0%)
	Total	34/91 (37.4%)	26/91 (28.6%)	22/91 (24.2%)

A Pearson's Chi Squared test was performed to evaluate the association between the presence of each of blepharospasm, corneal ulceration and uveitis, which identified a significant association between each of these three variables. Of the 34 cases with blepharospasm; 21 of these had corneal ulceration and 13 did not; of the 57 cases without blepharospasm; 5 of these had corneal ulceration and 52 did not (*χ* ^2^ = 29.305, df = 1, *p* < 0.001). Of the 34 cases with blepharospasm; 13 of these had signs of uveitis and 21 did not; of the 57 cases without blepharospasm; 9 of these had signs of uveitis and 48 did not (*χ* ^2^ = 5.853, df = 1, *p* = 0.016). Of the 26 cases with corneal ulceration; 10 of these had signs of uveitis and 16 did not; of the 65 cases that did not have corneal ulceration; 12 of these had signs of uveitis and 53 did not (*χ* ^2^ = 4.052, df = 1, *p* = 0.044).

A Pearson's Chi Squared test was performed to evaluate an association between each of blepharospasm, corneal ulceration and signs of uveitis with the outcome of enucleation, with a significant association identified between enucleation and both blepharospasm and uveitis, while there was no significant association between enucleation and corneal ulceration. Of the 34 cases with blepharospasm, 10 of these cases had enucleation and 24 did not; of the 57 cases without blepharospasm; 4 of these had enucleation and 53 did not (*χ* ^2^ = 8.204, df = 1, *p* = 0.004). Of the 22 cases with signs of uveitis; 9 of these had enucleation and 13 did not; of the 69 cases with no signs of uveitis, 5 of these had an enucleation and 64 did not (*χ* ^2^ = 14.521, df = 1, *p* < 0.001). Of the 26 cases with corneal ulceration, 7 of these had enucleation and 19 did not; of the 65 cases without corneal ulceration, 7 of these had enucleation and 58 did not (*χ* ^2^ = 3.723, df = 1, *p* = 0.054).

The topical treatment prior to presentation included combined corticosteroid and antibiotics (20/94, 21.3%), antibiotics (39/94, 41.5%), corticosteroids (13/94, 13.8%) and calcineurin inhibitors (15/94, 16.0%). Horses presenting to the RVC had received significantly more calcineurin inhibitors (*χ* ^2^ = 5.474, df = 1, *p* = 0.019) and horses presenting to Helsinki had received significantly more topical antibiotics (*χ* ^2^ = 13.800, df = 1, *p* < 0.001). Topical medication started or continued after assessment included combined corticosteroid and antibiotics (23/94, 24.5%), antibiotics (28/94, 29.8%), corticosteroids (38/94, 40.4%), and calcineurin inhibitors (44/94, 46.8%). Horses presenting to the RVC received significantly more combined corticosteroids and antibiotics (*χ* ^2^ = 4.652, df = 1, *p* = 0.031) and calcineurin inhibitors (*χ* ^2^ = 4.087, df = 1, *p* = 0.043), while horses presenting to Helsinki received significantly more topical corticosteroids (*χ* ^2^ = 9.701, df = 1, *p* = 0.002). Table 3 demonstrates further details regarding medication at presentation and after presentation for each institution.

**TABLE 3:** This table summarises the treatment of cases both before and after assessment at each institution.

	Medication started before presentation			Medication started after presentation		
	Total	RVC	Helsinki	Total	RVC	Helsinki
Topical combined steroid and antibiotic	20/94 (21.3%)	11/43 (25.6%)	9/51 (17.6%)	23/94 (24.5%)	15/43 (34.9%)	8/51 (15.7%)
Dexamethasone/neomycin/polymyxin B (*Maxitrol drops/ointment*, *Novartis*)	13/94 (13.8%)	11/43 (25.6%)	2/51 (3.9%)	22/94 (23.4%)	15/43 (34.9%)	7/51 (13.7%)
Dexamethasone disodium phosphate/chloramphenicol (*Oftan Dexa‐Chlora*, *Santen Oy*)	7/94 (7.4%)	0	7/51 (13.7%)	1/94 (1.1%)	0	1/51 (2.0%)
Topical steroid	14/94 (14.9%)	8/43 (18.6%)	5/51 (9.8%)	38/94 (40.4%)	10/43 (23.3%)	28/51 (54.9%)
Dexamethasone disodium phosphate 0.1% (*Maxidex*, *Novartis*) (*Oftan‐Dexa*, *Santen Oy*)	4/94 (4.3%)	3/43 (7.0%)	1/51 (2.0%)	12/94 (12.8%)	2/43 (4.7%)	10/51 (19.6%)
Prednisolone acetate 1% (*Pred Forte*, *AbbVie*)	9/94 (9.6%)	5/43 (11.6%)	4/51 (7.8%)	25/94 (26.6%)	8/43 (18.6%)	17/51 (33.3%)
Prednisolone acetate 0.5% (*Ultracortenol*, *Agepha*)	0	0	0	1/94 (1.1%)	0	1/51 (2.0%)
Unspecified steroid	1/94 (1.1%)	1/43 (2.3%)	0	0	0	0
Topical antibiotic	47/94 (50%)	9/43 (20.9%)	38/51 (74.5%)	30/94 (31.9%)	8/43 (18.6%)	22/51 (43.1%)
Fusidic Acid 1% (*Isathal*, *Dechra*)	4/94 (4.3%)	1/43 (2.3%)	3/51 (5.9%)	0	0	0
Chloramphenicol 0.5% drops, 0.01% ointment (*generic drops/ointment*), (*Oftan‐Akvakol/Oftan‐Chlora Santen Oy*)	30/94 (31.9%)	5/43 (11.6%)	25/51 (49.0%)	24/94 (25.5%)	7/43 (16.3%)	17/51 (33.3%)
Ofloxacin 0.3% (*Exocin*, *AbbVie*)	6/94 (6.4%)	0	6/51 (11.8%)	2/94 (2.1%)	1/43 (2.3%)	1/51 (2.0%)
Moxifloxacin 0.5% (*Vigamox*, *Novartis*)	1/94 (1.1%)	0	1/51 (2.0%)	3/94 (3.2%)	0	3/51 (5.9%)
Ciprofloxacin 0.3% (*Ciloxan*, *Novartis*)	3/94 (3.2%)	3/43 (6.9%)	0	0	0	0
Terramycin/Polymixin B (*Terra‐Poly Vet*, *Zoetis*)	1/94 (1.1%)	0	1/51 (2.0%)	0	0	0
Chlortetracycline 1% (*Aureomycin*, *Alpharma*)	0	0	0	1/94 (1.1%)	0	1/51 (2.0%)
Unspecified antibiotic	2/94 (2.1%)	0	2/51 (3.9%)	0	0	0
Topical calcineurin inhibitor	16/94 (17.0%)	12/43 (27.9%)	4/51 (7.8%)	44/94 (46.8%)	25/43 (58.1%)	19/51 (37.3%)
Ciclosporin 0.2% (*Optimmune*, *MSD*)	14/94 (14.9%)	11/43 (25.6%)	3/51 (5.9%)	42/94 (44.7%)	24/43 (55.8%)	18/51 (35.3%)
Ciclosporin 0.1% (*compounded/Ikervis*, *Santen Oy*)	1/94 (1.1%)	0	1/51 (2.0%)	0	0	0
Ciclosporin 1.0% (*compounded*)				1/94 (1.1%)	0	1/51 (2.0%)
Tacrolimus 0.02% (*compounded*, *Bova*)	1/94 (1.1%)	1/43 (2.3%)	0	1/94 (1.1%)	1/43 (2.3%)	0
Topical non‐steroidal anti‐inflammatory	0	0	0	8/94 (8.5%)	3/43 (7.0%)	5/51 (9.8%)
Ketorolac 0.5% (*Acular*, *AbbVie*)	0	0	0	2/94 (2.1%)	2/43 (4.7%)	0
Bromfenac 0.09% (*Yellox*, *Bausch & Lomb*)				6/94 (6.4%)	1/43 (2.3%)	5/51 (9.8%)
Systemic non‐steroidal anti‐inflammatory	31/94 (33.0%)	13/43 (30.2%)	18/51 (35.3%)	29/94 (30.9%)	17/43 (40.0%)	12/51 (23.5%)
Suxibuzone (*Danilon*, *Animalcare*)	3/94 (3.2%)	3/43 (6.9%)	0	7/94 (7.4%)	7/43 (16.3%)	0
Phenylbutazone (*Equipalazone*, *Dechra*)	6/94 (6.4%)	6/43 (14.0%)	0	3/94 (3.2%)	3/43 (6.9%)	0
Flunixin (*Finadyne*, *MSD*)	17/94 (18.0%)	4/43 (9.3%)	13/51 (25.5%)	19/94 (20.2%)	7/43 (16.3%)	12/51 (23.5%)
Meloxicam (*Metacam*, *Boehringer Ingelheim*)	4/94 (4.3%)	0	4/51 (7.8%)	0	0	0
Firocoxib (*Equioxx*, *Boehringer Ingelheim*)	1/94 (1.1%)	0	1/51 (2.0%)	0	0	0
Topical antifungal	3/94 (3.2%)	2/43 (4.7%)	1/51 (2.0%)	1/94 (1.1%)	1/43 (2.3%)	0
Voriconazole 1% (*VFend 200 mg*, *Pfizer*)	1/94 (1.1%)	0	1/51 (2.0%)	1/94 (1.1%)	1/43 (2.3%)	0
Silver sulfadiazine 1% (*Flamazine*, *Smith & Nephew*)	1/94 (1.1%)	1/43 (2.3%)	0	0	0	0
Unspecified antifungal	1/94 (1.1%)	1/43 (2.3%)	0	0	0	0
Other						
Serum	5/94 (5.3%)	1/43 (2.3%)	4/51 (7.8%)	3/94 (3.2%)	2/43 (4.7%)	1/51 (2.0%)
Atropine 1% (*Minims Atropine*, *Bausch & Lomb*)	21/94 (22.3%)	9/43 (20.9%)	12/51 (23.5%)	11/94 (11.7%)	5/43 (11.6%)	6/51 (11.8%)
Aciclovir 3% (*generic formulation*)	0	0	0	1/94 (1.1%)	1/43 (2.3%)	0
Systemic doxycycline injectable 10% (*Engemycin*, *MSD*)	0	0	0	1/94 (1.1%)	1/43 (2.3%)	0

**FIGURE 2:**
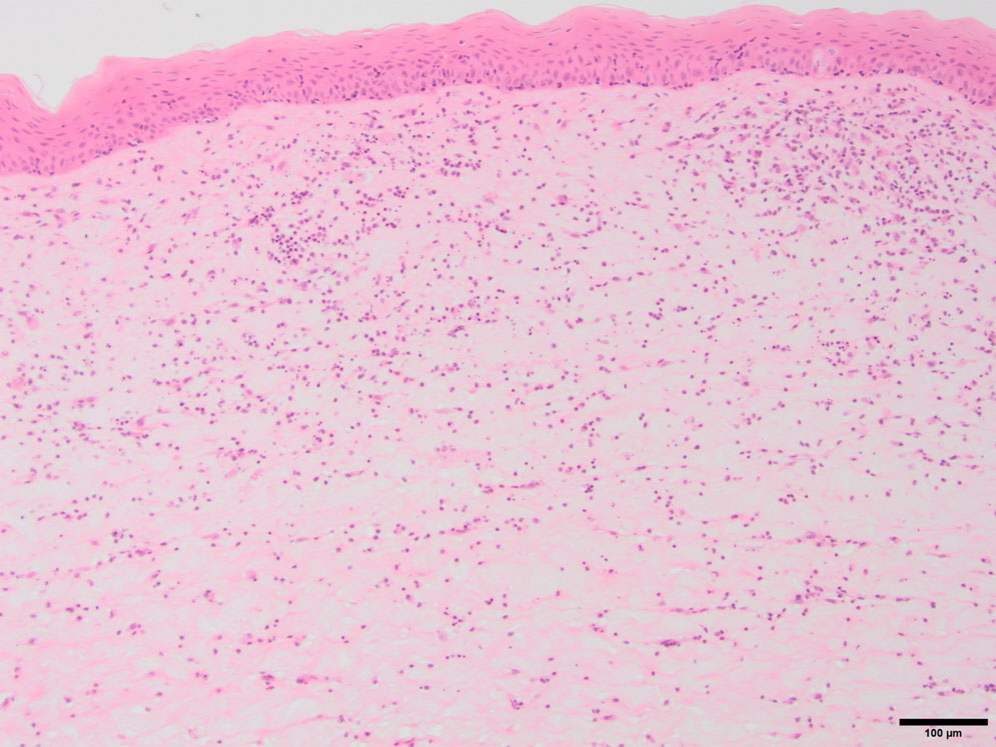
Microscopic image from cornea of a typical immune mediated keratitis case. The corneal epithelium is largely unremarkable. The stroma is diffusely expanded by moderate to marked oedema, and is infiltrated by moderate numbers of lymphocytes and plasma cells, occasionally forming small aggregates and contains leukocytoclastic cellular debris. H&E, ×100.

Corneal cytology was documented in 28/94 (29.8%) of eyes; these reported epithelial cells only (*n* = 21), epithelial cells and neutrophils (*n* = 5), epithelial cells, neutrophils and low numbers of cocci bacteria (*n* = 1), epithelial cells, neutrophils, lymphocytes and plasma cells (*n* = 1). Corneal culture and sensitivity was documented in 14/94 (14.9%) of eyes; these reported no growth (*n* = 11), low volume mixed growth and presumed contamination (*n* = 2), and *Streptococcus* sp. (*n* = 1). Histopathology was performed on eight samples; six globes and two keratectomy samples, all of which were considered supportive for inflammatory keratitis with no identified cause (Figure 2). Both keratectomy samples found no micro‐organisms, one demonstrated a marked lymphoplasmacytic infiltration of the stroma, and one neutrophilic infiltration of the stroma with multifocal mineralisation. Of the six globes examined, none demonstrated presence of micro‐organisms. The histopathology findings were summarised as moderate multifocal lymphoplasmacytic stromal keratitis (*n* = 1); moderate neutrophilic stromal keratitis with focal corneal ulceration (*n* = 1); moderate predominantly neutrophilic and less lymphoplasmacytic stromal keratitis (*n* = 1); marked predominantly neutrophilic and less lymphoplasmacytic infiltration of the corneal stroma and base of the iris (*n* = 1); marked corneal oedema with moderate lymphoplasmacytic infiltration between Descemet's membrane and the endothelium and lymphoplasmacytic infiltration of the iris around the iridocorneal angle (*n* = 1); and corneal ulceration with marked infiltration of the stroma with neutrophils, lymphocytes and plasma cells, with mild neutrophilic and lymphoplasmacytic infiltration of the iris and ciliary body (*n* = 1). Of the eight eyes with histopathology supporting IMMK diagnosis the following signs of uveitis were documented; one had low IOP, ‘marked’ miosis and a ‘trace’ of flare; one had a normal IOP, ‘moderate’ miosis and ‘moderate flare’; one had a normal IOP, unclassified miosis and ‘minimal’ flare; the last had low IOP, ‘slight’ miosis and no flare.

Surgical procedures or euthanasia performed either at presentation or throughout the treatment period are summarised in Table 4. Of the 16 horses that had episcleral ciclosporin A implants placed, only one case returned for repeat implants after 745 days, four cases were enucleated after 4, 39, 52 and 344 days, and the remainder were lost to follow‐up. The mean follow‐up time for cases that received episcleral ciclosporin implants was 224.8 ± 285.8 days.

**TABLE 4:** This table summarises the surgical procedures performed both at initial presentation and at any time through the treatment period, and whether surgical procedures resulted in subsequent enucleation.

Procedure type	At presentation	At any time	Subsequent enucleation
Euthanasia	4	5	N/A
Enucleation	1	14	N/A
Episcleral CsA^a^ implants	3	11	1
Keratectomy and CPG^b^	3	3	‐
Keratectomy and CPG^b^ with episcleral CsA^a^ implants	1	3	2
Keratectomy and episcleral CsA^a^ implants	0	2	1
Diamond burr debridement	2	3	1

^a^ Ciclosporin A.

^b^ Conjunctival pedicle graft.

Superficial keratectomy was carried out in two cases, one case had episcleral ciclosporin A implants at the time and had these implants replaced 2 years later, with a successful long‐term follow‐up of 1061 days with only ciclosporin A implants as medication. The other was enucleated 5 weeks later due to a heavily infiltrated corneal ulcer. Five cases in this series had a keratectomy and conjunctival graft; three cases were lost to follow‐up from 2 to 8 weeks after surgery; one of these had episcleral ciclosporin A implants placed and two were on topical medications. The topical medications these two cases were prescribed at the last available re‐examination was ongoing ciclosporin A and a course of corticosteroids (combined dexamethasone/neomycin/polymyxin B for one case and prednisolone acetate for the other) tapering over a month before discontinuation. The other two cases with a keratectomy and conjunctival graft were enucleated; one 3 days later and one 3 weeks later due to further deterioration; the first case had a concern for leakage of aqueous humour and a marked fibrinous uveitis, the second had a stromal abscess develop adjacent to the surgical site and marked uveitis.

Diamond burr debridement was performed in three horses that developed corneal mineralisation. In two horses this was performed at the time of presentation; both of these horses had corneal ulceration and it was considered that the corneal mineralisation was inhibiting ulcer healing; and in one horse it was performed during the follow‐up period. One horse had subsequent enucleation after 571 days of follow‐up; the owner had been recommended a keratectomy and this had been declined. Two horses retained their eyes to the last follow‐up time of 25 days and 395 days.

Enucleation was performed at any timepoint during follow‐up in 14/94 (14.9%) horses; the IMMK subtype of these cases was unrecorded (*n* = 2/14, 14.3%), anterior stromal (*n* = 7/14, 50%), mid‐stromal (*n* = 3/14, 21.4%) and endothelial (*n* = 2/14, 14.3%). The decision to enucleate was attributed to the IMMK pathology in all cases; with 7/14 occurring due to poor response to medical treatment, 5/14 occurring after prior surgical intervention and 2/14 due to complications (one stromal abscess and one infected corneal ulcer). Euthanasia was performed in 4/94 (4.3%) of horses during the study follow‐up; of these, three were euthanised due to the owner's preference after diagnosis of IMMK, and one was due to concern for long‐term vision as the fellow eye displayed phthisis bulbi. Clinical records available beyond the last available ophthalmic examination indicated euthanasia in another eight cases; seven of these were for unrelated reasons and one was due to recurrent corneal ulceration secondary to IMMK.

The median follow‐up time was 27 days (range 0–1430 days) and was available for 66 horses with 18/66 horses having either been enucleated or euthanised at the last exam. Of the remaining 48 horses with follow‐up available, all were visual, 4/48 (8.3%) were painful, 16/48 (33.3%) had mild scarring due to IMMK, 1/48 (2.1%) had mild scarring due to unrelated corneal trauma and 12/48 (25.0%) had moderate scarring due to IMMK. The majority of horses, 37/48 (77.1%) were on ongoing therapy, although 5/48 (10.4%) were only receiving ciclosporin A via an episcleral implant and did not need additional topical medication. The IMMK classifications of those managed with ciclosporin A implants alone were epithelial (2/5); anterior stromal (1/5); endothelial (1/5) and unrecorded (1/5). In 11/48 (22.9%) all medication had been successfully discontinued with a mean number of days of controlled clinical signs without medication of 538 (±448). The IMMK classifications of those entirely off medication were epithelial (2/12); anterior stromal (5/12); mid‐stromal (3/12) and unrecorded (1/12).

Of the 34 horses without incidental ulceration that presented with blepharospasm, 10/34 (29.4%) subsequently had an enucleation. The odds ratio of having an enucleation with the presence of blepharospasm versus no blepharospasm was 5.521 (95% confidence interval [CI] 1.573–19.382, *p* = 0.008). Of the 26 horses that presented with corneal ulceration due to IMMK, 7/26 (26.9%) subsequently had enucleation. The odds ratio of having an enucleation with the presence of corneal ulceration versus no corneal ulceration was 3.053 (95% CI 0.949–9.823, *p* = 0.061). Of the 22 horses that presented with signs of uveitis, 9/22 (40.9%) subsequently had enucleation. The odds ratio of having enucleation with the presence of uveitis versus without uveitis was 8.862 (95% CI 2.551–30.783, *p* < 0.001).

Of the 45 horses without incidental ulceration and with follow‐up available there was no significant association between the need for ongoing medication and the presence of blepharospasm (OR 2.087, 95% CI 0.381–11.417), corneal ulceration (OR 1.071, 95% CI 0.230–4.990) or signs of uveitis (OR 1.0, 95% CI 0.173–5.795).

## 4 DISCUSSION

This study is the first European‐based large case series on IMMK, providing a more objective understanding of the disease presentation in Europe and allowing comparison of features of IMMK with findings in the USA which has been described in previous publications. This study demonstrated that overall, the two unrelated populations in the UK and Finland had very similar presentation and outcome, suggesting that the disease manifestation across Northern Europe is similar. The difference in corneal scarring between the two populations is likely due to slight discrepancies in how clinical parameters were recorded and inter‐observer bias at the time of care. Overall, the study highlighted some differences between the results of this study and previously published descriptions of IMMK.

The previous subjective report of IMMK in the UK describes the possibility of mild–moderate blepharospasm with the epithelial and anterior stromal forms of the disease, and occasionally with the mid‐stromal form if oedematous corneal bullae form and subsequently rupture.^1^ This study supports this finding with over a third of cases presenting with blepharospasm.

Fluorescein retention has been previously described as a feature of IMMK by Matthews (2000), both as retention of fluorescein in the interstices between islands of abnormal epithelium in the epithelial form, and as a consequence of ruptured bullae secondary to corneal oedema in the mid‐stromal and endothelial forms.^3^ In the case series by Gilger et al. (2005) fluorescein uptake was not identified in any horse.^2^ This case series has identified corneal ulceration across all four sub‐types of IMMK in 28.6% of all cases. The retrospective nature of this study limits qualitative understanding of the degree of fluorescein retention; it is likely that there is a spectrum from mild, erosive uptake to complete loss of epithelium. The presence of corneal ulceration therefore should not exclude a diagnosis of IMMK. Further studies looking into more specific ophthalmic findings may clarify the relevance of certain changes such as corneal degeneration or mineralisation that can occur with chronicity of both disease and topical medication.^4^, ^6^, ^13^, ^32^ The timeframe in which a horse is examined may impact the clinical signs apparent at the examination, and it is possible that these degenerative changes secondary to chronic disease or chronic topical medication may predispose to further ocular abnormalities, for example, corneal ulceration. It may, therefore, be challenging to determine which clinical findings are directly attributed to the disease process, and what may be a secondary effect of topical medication.

In the case series by Gilger et al. (2005) the mean duration of signs was 11.8 months prior to referral, whereas the mean duration in this study was only 7 weeks. This may indicate that since 2005 there has been improved recognition of IMMK and the benefit of referring cases to an ophthalmologist for evaluation with a slit lamp biomicroscope and treatment planning. The case series by Gilger et al. (2005) identified no epithelial cases, a similar proportion of anterior stromal (50%) and mid‐stromal (27%) and a greater proportion of endothelial (23%) sub‐type compared with our population. This variation in the sub‐type of IMMK might further support the idea of variations in the inciting causes between the USA and the UK. With the more recent description of heterochromic iridocyclitis and keratitis (HIK) it is possible that some of the cases previously defined as ‘endothelial’ might now be classified as HIK in the context of current literature.^10^

The significantly lower intraocular pressure in some of the affected eyes is a novel finding. Previously, in the case series by Gilger et al. (2005) normal intraocular pressures were described in all cases, although in that study the lack of uveitis was an inclusion criteria, and it describes that no horses presented with aqueous humour flare or miosis.^2^ Matthews (2000) described no evidence of intraocular inflammation in any classification of IMMK, which is in contrast to our study where some signs of uveitis are present in 24% of cases across all subtypes of IMMK.^1^, ^2^ The presence of corneal pathology could have impacted the measurement of intraocular pressure, as this has been previously documented in canine eyes.^22^ The effect of corneal pathology on the IOP reading could have introduced bias and artificially inflated the number of horses with reduced intraocular pressure. The statistical difference in IOP is probably not a clinically relevant difference but highlights the possibility of mild uveitis in some cases. Signs of uveitis were documented in 22 cases and may be a form of ‘reflex uveitis’ secondary to the corneal disease; only 10/22 were documented to have corneal ulceration, suggesting that corneal inflammation may be sufficient to trigger a reflex uveitis.

The differences in medications started both before and after assessment between the two institutions is likely to be multifactorial; there are differences in guidance to vets about medication decision‐making and different licensing for medications. The higher number of antibiotics prescribed to horses in Finland suggests that more of the referring vets had either not reached the correct diagnosis or had not recommended the correct treatment. In the authors' opinion the uncertainty regarding the correct diagnosis together with the reported increased prevalence of fungal infections in Finland compared with the United Kingdom may contribute to prescribing antimicrobial medications and referring the case.^33^, ^34^ The higher number of horses started on combined topical corticosteroid and antibiotics in the RVC population may be driven by the lack of availability of a corticosteroid‐only ointment formulation in the UK.

The need for ongoing therapy can be a challenge for the owners and can incur restrictions on the ability of the horse to comply with competition regulations, as, for example, topical corticosteroids would be considered a controlled medication by the Fédération Équestre Internationale.^15^ In this study 77.1% of horses required ongoing medication at the last examination. This information is valuable as it allows for clear communication with the clients at the time of diagnosis allowing for management of expectation. It might also encourage horse owners to commit to positive training techniques for applying ocular medication at the outset to improve long‐term compliance. In addition, clients may opt for surgical interventions such as a keratectomy or episcleral ciclosporin A implants at the outset knowing that medical management alone is unlikely to be curative. The odds of needing ongoing medication were not significantly increased by the presence of blepharospasm, corneal ulceration or uveitis and therefore clinical signs can unfortunately not be used as a predictor for needing long‐term medication. The more recent use of episcleral ciclosporin A implants has offered the potential to medicate these cases without the need for daily topical administration. A previous study identified excellent control of the disease with the use of episcleral ciclosporin A implants in the anterior stromal and the endothelial groups, but not the mid‐stromal groups.^9^ In our study, five cases were controlled with ciclosporin A implants only, and none of these had mid‐stromal IMMK. Previous studies have suggested good tolerance of silicone ciclosporin A implants, with an estimated duration of action of 12–18 months.^9^, ^35^, ^36^ Alternative novel therapies that might reduce the need for ongoing topical treatment include sub‐conjunctival injection of mesenchymal stem cells and the use of indocyanine green intrastromal injections followed by irradiation.^12^, ^37^ However, long‐term results for these therapies still need to be investigated in larger groups of horses.

The pathogenesis of IMMK is poorly understood but given the reported response to topical corticosteroids and ciclosporin A, an aberrant immune reaction to a corneal insult or foreign antigen, either genuine or misidentified by the immune system, is considered highly likely.^11^, ^14^, ^32^ It has been suggested that the epithelial form might be associated with viral or fungal antigens residing in the superficial cornea and causing chronic immune stimulation.^3^, ^32^ If this is true then one would expect to see a pattern in the type of IMMK classification based on prevalence of fungal keratitis; a wider study including more geographical locations would be needed to investigate this further. Keratomycoses are reported to be more prevalent in warm, humid locations,^4^, ^38^ and various studies from different geographical locations have reported distinct seasonality patterns and differences in the most prevalent fungi cultured.^39^, ^40^, ^41^, ^42^ Studies have so far failed to prove the presence of microorganisms within corneal specimens from keratectomy, but in vivo confocal microscopy has demonstrated large numbers of antigen‐presenting cells surrounding unidentifiable structures in the subepithelial and anterior stroma, which could be fragments of a microorganism or foreign structure.^13^ Superficial keratectomy is postulated to remove these offending antigens. In a previous US case series superficial keratectomy and conjunctival grafts were curative in four horses with anterior stromal IMMK and one horse with mid‐stromal IMMK with no further ongoing medication required.^2^ An abstract published by Pate et al. (2009) described the successful treatment of 13 cases with keratectomy alone.^43^ Superficial keratectomy either with or without graft in this study failed to control the disease in half of the cases, and the other half were lost to follow‐up. One could presume those lost to follow‐up were successful, but even if this is the case superficial keratectomy does not appear to be as successful in Europe as reported in a small number of cases in the USA. A more recent European‐based report describes superficial keratectomy alone in four cases; in one case the IMMK recurred and two cases had complications after surgery, one of these then required a conjunctival pedicle graft.^44^ This difference in the success of treatment by keratectomy could suggest a possible difference in the underlying pathophysiology of the superficial stromal cases between Europe and the USA, however, a larger number of cases with a strict inclusion criteria regarding the details and the stage of the disease are required.

In this study, enucleation was the final outcome in 14.9% of eyes, which is slightly higher than the 9% previously reported.^2^ Interestingly, none of the cases that had enucleation were classified as the epithelial subtype; perhaps reflecting that this form is more responsive to treatment. The increased odds of enucleation in cases presenting with blepharospasm or signs of uveitis may suggest that these cases have a more intense inflammatory process, which may be more likely to be refractory to medical management. The underlying decision‐making points for each case undergoing enucleation were difficult to establish in the retrospective data, but all enucleations were attributed to the IMMK disease process and most were due to poor treatment response. As this study investigates a referral population it is possible that there is a bias towards complicated cases that are refractory to medical therapy initiated prior to referral, which may make these cases more likely to result in enucleation.

This study provides clinicians with a framework to discuss potential outcomes with the owner; the majority of cases will be comfortable and visual but ongoing treatment is likely needed and a degree of scarring can be expected. Some horses simply do not tolerate topical medication, and the episcleral ciclosporin A implants alone were only successful in controlling 10.4% of cases in this study. Owners with particularly head shy horses may feel that the treatment regime is detrimental to their relationship with the horse and therefore not pursue treatment. Owners of sport horses that require excellent visual acuity may consider the risk of corneal scarring and potential impact on vision in their decision‐making process.

The retrospective nature of this study is one of the key limitations, as clinical records had some missing data and there was a likely inter‐observer variation in the initial recording of clinical notes. Diagnosis was reached on clinical evaluation only in the majority of cases and this is likely a multifactorial limitation; cytology data may be missing from the clinical records, clients may have declined the additional cost of histopathology, or clinicians and/or the clients may have elected against cytology on non‐ulcerated corneas as this may have then created a corneal ulcer which can then limit the initial treatment options and incur additional expense for the client to have an additional re‐examination. In particular, the small number of samples available for histopathology (most likely due to financial implication of submitting the samples) is a major limitation, as this is the only way to confirm the diagnosis. Future prospective studies could be designed with cytology and histopathology as required inclusion criteria. The data from each hospital spans a different time frame, which allowed for a similar number of cases between the two groups but may introduce compounding factors, as the understanding of IMMK and its treatment might have evolved over time. As discussed, the interpretation of the signs of uveitis, particularly that of low intraocular pressure, are a potential source of bias. The short follow‐up time limits interpretation of the long‐term findings and introduces bias with regards to corneal scarring, as this can take a long time to remodel and may appear more severe in cases with shorter follow‐up. The RVC data also includes a small number of clinical examination findings provided by Diplomates of the European College of Equine Internal Medicine prior to the formation of a specialised ophthalmology service, which may result in different interpretations of clinical features of the disease.

## 5 CONCLUSION

Blepharospasm, corneal ulceration and signs of uveitis can occur in all sub‐types of IMMK. Patients presenting with either blepharospasm or signs of uveitis have significantly increased odds of enucleation. The presentation and outcomes of IMMK in two geographically distinct regions of Europe is similar, but conflicts with some features of the published USA disease description.

## FUNDING INFORMATION

Not applicable.

## CONFLICT OF INTEREST STATEMENT

The authors declare no conflicts of interest.

## AUTHOR CONTRIBUTIONS

**Juliette F. Preston:** Writing – original draft; investigation; formal analysis; data curation; writing – review and editing; conceptualization. **Minna P. Mustikka:** Conceptualization; investigation; writing – review and editing; methodology. **Simon L. Priestnall:** Writing – review and editing; investigation. **Bettina Dunkel:** Conceptualization; methodology; writing – review and editing; supervision. **Maria‐Christine Fischer:** Conceptualization; writing – review and editing; methodology; supervision.

## DATA INTEGRITY STATEMENT

Juliette F. Preston had full access to all the data in the study and takes responsibility for the integrity of the data and the accuracy of data analysis.

## ETHICAL ANIMAL RESEARCH

Data collected from RVC falls under the RVC Social Science Research Ethical Review Board, which does not require specific ethical approval for purely retrospective research using clinical data. Data collected from Helsinki had appropriate ethical approval, reference Statement 19/2021.

## INFORMED CONSENT

All owners of animals admitted to the hospitals are informed that clinical data are used for research purposes.

### PEER REVIEW

The peer review history for this article is available at https://www.webofscience.com/api/gateway/wos/peer-review/10.1111/evj.14213.

## Data Availability

The data that support the findings of this study are available from the corresponding author upon reasonable request: Open sharing exemption granted by editor for this descriptive retrospective clinical report.
